# Supplementary material for: Diagnostic Accuracy and economic value of a Tiered Assessment for Fetal Alcohol Spectrum Disorder (DATAforFASD): Protocol
Source: BMJ Open. 2023 Aug 16;13(8):e071004. doi: 10.1136/bmjopen-2022-071004 (PMC10432646; doi:10.1136/bmjopen-2022-071004)
Supplement: Author's manuscript [file bmjopen-2022-071004.draft_revisions.pdf]

BMJ Open

BMJ Open is committed to open peer review. As part of this commitment we make the peer review history of every article we publish publicly available.

When an article is published we post the peer reviewers' comments and the authors' responses online. We also post the versions of the paper that were used during peer review. These are the versions that the peer review comments apply to.

The versions of the paper that follow are the versions that were submitted during the peer review process. They are not the versions of record or the final published versions. They should not be cited or distributed as the published version of this manuscript.

BMJ Open is an open access journal and the full, final, typeset and author-corrected version of record of the manuscript is available on our site with no access controls, subscription charges or pay-per-view fees (http://bmjopen.bmj.com).

If you have any questions on BMJ Open's open peer review process please email info.bmjopen@bmj.com

BMJ Open

Diagnostic Accuracy and economic value of a Tiered Assessment for Fetal Alcohol Spectrum Disorder (DATAforFASD): Protocol

Journal:	*BMJ Open*
Manuscript ID	bmjopen-2022-071004
Article Type:	Protocol
Date Submitted by the Author:	12-Dec-2022
Complete List of Authors:	Shanley, Dianne; Griffith University, Menzies Health Institute of Queensland/School of Applied Psychology<br>Zimmer-Gembeck, Melanie ; Griffith University, Menzies Health Institute of Queensland/School of Applied Psychology<br>Wheeler, Amanda; Griffith University, Menzies Health Institute of Queensland; The University of Auckland, Faculty of Medical & Health Sciences<br>Byrnes, Joshua; Griffith University, Menzies Health Institute of Queensland/School of Medicine and Dentistry<br>Ware, Robert; Griffith University, Menzies Health Institute of Queensland<br>Liu, Wei; Griffith University, Menzies Health Institute of Queensland<br>Simcock, Gabrielle; Griffith University, Menzies Health Institute of Queensland<br>White, Codi; Griffith University, Menzies Health Institute of Queensland<br>Horton, Sarah; Gidgee Healing, Family Health<br>Page, Marjad; Kambu Aboriginal and Torres Strait Islander Corporation for Health<br>Shelton, Doug; Gold Coast Hospital and Health Service; Griffith University, Menzies Health Institute of Queensland/School of Medicine and Dentistry<br>Till, Haydn; Gold Coast Hospital and Health Service; Griffith University, Menzies Health Institute of Queensland/School of Medicine and Dentistry<br>Mills, Ianthe; Sunshine Coast Hospital and Health Service<br>Hislop, Carly; Townsville Hospital and Health Service, Child Development Service<br>Harris, Katrina ; Monash Health, Victorian Fetal Alcohol Service; Monash University, Department of Paediatrics<br>Crichton, Alison; Monash Health, Victorian Fetal Alcohol Service; Monash University, Department of Paediatrics<br>Reid, Natasha; The University of Queensland,<br>Reilly, Sheena; Griffith University,<br>Moritz, Karen; The University of Queensland, ; The University of Queensland Child Health Research Centre<br>Walsh, K.; Queensland University of Technology,<br>Rundle-Thiele, Sharyn; Griffith University,<br>Hawkins, Erinn; Griffith University, School of Applied Psychology/Menzies Health Institute of Queensland
Keywords:	PRIMARY CARE, HEALTH ECONOMICS, Community child health <

	PAEDIATRICS, Developmental neurology & neurodisability < PAEDIATRICS, Health Services Accessibility

SCHOLARONE™
Manuscripts

Title: Diagnostic Accuracy and economic value of a Tiered Assessment for Fetal Alcohol Spectrum Disorder (DATAforFASD): Protocol

Running Head: DATAforFASD Protocol

Dianne C. Shanley[a,b]*, Melanie J. Zimmer-Gembeck[a,b], Amanda J Wheeler[a,c], Joshua Byrnes[a,d], Robert S Ware[a], Wei Liu[a], Gabrielle Simcock[a], Codi White[a], Sarah Horton[g], Marjad Page[a,h], Doug Shelton[a,d,e], Haydn Till[a,d,e], Ianthe Mills[f], Carly Hislop[i], Katrina Harris[j,k], Ali Crichton[j,k], Natasha Reid[l], Sheena Reilly[a], Karen M. Moritz[m], Kerryann Walsh[n], Sharyn Rundle-Thiele[o], and Erinn Hawkins[a,b]

[a] Menzies Health Institute of Queensland, Griffith University, Australia
[b] School of Applied Psychology, Griffith University, Australia
c Faculty of Medical & Health Sciences, University of Auckland, NZ
d School of Medicine and Dentistry, Griffith University, Australia
e Community Child Health, Gold Coast Hospital and Health Service, Australia
f Child and Adolescent Unit, Sunshine Coast Hospital and Health Service, Australia
g Family Health, Gidgee Healing, Australia
h Executive team, Kambu Aboriginal and Torres Strait Islander Corporation for Health, Australia
i Child Development Service, Townsville Hospital and Health Service, Australia
j Victorian Fetal Alcohol Service, Monash Children's Hospital, Australia
k Department of Paediatrics, Monash University, Australia
l Child Health Research Centre, University of Queensland, Australia
m Faculty of Medicine, University of Queensland, Australia
n School of Early Childhood and Inclusive Education, Queensland University of Technology, Australia
o Social Marketing@Griffith, Griffith University, Australia

***Corresponding author**

Dianne C. Shanley
Menzies Health Institute Queensland, School of Applied Psychology
Griffith University, Gold Coast Campus
Parklands Drive, QLD 4222
Australia
d.shanley@griffith.edu.au

Word count: 3,945

For peer review only - http://bmjopen.bmj.com/site/about/guidelines.xhtml

ABSTRACT

Introduction: Australian practices for diagnosing fetal alcohol spectrum disorder (FASD) are lengthy and require specialist expertise. Specialist teams are based in urban locations; they are expensive and have prolonged waitlists. Innovative, flexible solutions are needed to ensure First Nations children living in rural/remote communities have culturally appropriate and equitable access to timely diagnosis and support. This study compares the accuracy of rapid assessments (index tests) that can be administered by a range of primary healthcare practitioners to specialist standardised FASD assessments (reference tests). The cost-efficiency of index tests will be compared to reference tests.

Methods and analysis: At least 200 children aged 6-16 years at-risk of FASD will be recruited across 8 study sites [redacted for blind peer review]. Following STARD diagnostic accuracy guidelines, all children will complete index and reference tests. Diagnostic accuracy statistics (including receiver operating curves, sensitivity, specificity, positive and negative predictive values, and likelihood ratios) will identify whether rapid assessments can accurately identify: (a) the presence of a FASD diagnosis and (b) impairment in each neurodevelopmental domain, compared to comprehensive assessments. Direct and indirect healthcare costs for index tests compared to reference tests will be collected in primary healthcare and specialist settings.

Ethics and dissemination of results: [redacted for blind peer review] Hospital and Health Service Human Research Ethics Committee (HREC/20/XXXX/63173); [redacted for blind peer review] Human Research Ethics Committee (2020/743).

For peer review only - http://bmjopen.bmj.com/site/about/guidelines.xhtml

Results will assist in validating the use of index tests as part of a tiered neurodevelopmental assessment process that was co-designed with First Nations community and primary healthcare practitioners. Outcomes will be summarised and provided to participating practitioners and sites and disseminated to community health services and consumers. Findings will be presented at national and international conferences and published in peer-reviewed journals.

Registration details: ACTRN12622000498796 (29 March 2022)

Key words: fetal alcohol spectrum disorder; primary care; Indigenous Health; neurodevelopmental disorder; diagnostic tests and procedures; cost-benefit analysis; co-design

For peer review only

For peer review only - http://bmjopen.bmj.com/site/about/guidelines.xhtml

STRENGTHS AND LIMITATIONS OF THIS STUDY

- DATAforFASD is the first large, prospective, multi-site trial to compare comprehensive assessments to rapid assessments across nine domains of neurodevelopment so that we can better understand the accuracy of using rapid assessments to screen and diagnose neurodevelopmental disorders like FASD.
- A sample size of at least 200 children is uncommon in FASD research and may prove to be challenging, because completing gold-standard comprehensive assessments on 9 neurodevelopmental domains requires around 2 days of multi-disciplinary specialist (paediatrician/allied health) time per child.
- The rapid assessment fits within a co-designed tiered assessment process that is responsive to cultural protocols and limited health care resources in remote regions. It includes the Rapid Neuro-developmental Assessment, an observational functional neurodevelopmental assessment, and the Behavioural Assessment Scale for Children, 3rd Ed.
- This project follows STARD guidelines, providing the methodological rigor necessary to assess the diagnostic accuracy of rapid assessments in identifying FASD, as well as their accuracy in identifying neurodevelopmental impairment across 9 domains; the cost benefit of using rapid assessment will be explored for families and health services.
- Establishing valid, accurate rapid assessments will enable: (a) early detection of at-risk children, particularly in rural and remote communities where access to care is inequitable; (b) non-specialist care providers (health, education, youth justice, child protection) to confidently contribute to information needed for diagnostic purposes; (c) enhanced triage, resulting in better quality referrals to specialists; (d) the use of rapid assessments for diagnostic purposes, potentially addressing a systemic

For peer review only - http://bmjopen.bmj.com/site/about/guidelines.xhtml

healthcare challenge by offsetting the longest most costly assessment process for the most prevalent preventable neurodevelopmental disorder.

For peer review only

For peer review only - http://bmjopen.bmj.com/site/about/guidelines.xhtml

INTRODUCTION

Fetal alcohol spectrum disorder (FASD) affects between 1-5% of Australian children, and up to 19% in some remote Australian communities, which is higher than any other neurodevelopmental disorder.[1-4] To identify FASD, current Australian guidelines recommend comprehensive diagnostic assessments (reference tests) across ten neurodevelopmental domains, delivered by multi-disciplinary teams. These assessments are primarily available in Australia at specialist services, making this a lengthier and more costly diagnostic process than any other neurodevelopmental disorder.[5] Current waitlists for Australian children suspected of FASD are up to 3 years at some health services. Overall, specialist services are only seeing a small proportion of suspected cases and children from rural and remote regions are left behind.[6, 7]

To meet the demand for diagnosis, our Australian health system either needs an inordinate number of specialists and multidisciplinary clinics, or we need to think differently about how to diagnose FASD. Our health system is unlikely to be able to resource the longest, most costly assessment process for the most prevalent neurodevelopmental disorder in children, but the use of valid and reliable rapid assessment tools could leverage capacity within primary care, increasing vulnerable children's access to diagnosis.

In this project we will conduct a two-phase study to: (i) determine the diagnostic accuracy of rapid neurodevelopment assessments (index tests) that can be used to detect FASD in primary healthcare (Phase I);[8] and (ii) examine the economic benefits of leveraging primary healthcare to support specialist practitioners in the diagnosis of FASD, in terms of cost for families and healthcare systems (Phase II).

For peer review only - http://bmjopen.bmj.com/site/about/guidelines.xhtml

METHODS AND ANALYSIS

Phase I: Diagnostic accuracy

Study design

A prospective diagnostic accuracy study (following STARD guidelines) will compare index tests to reference tests for 9 of 10 neurodevelopmental domains commonly used by specialist, multi-disciplinary teams when diagnosing FASD (brain structure domain is not included).[9]

Primary and Secondary Outcomes

The first primary outcome will determine the diagnostic accuracy of identifying FASD with index tests compared to reference tests. The second primary outcome will determine the accuracy of identifying impairment within each domain assessed. It is hypothesised that the rapid assessments (index tests) will have good sensitivity and specificity when identifying: (a) the presence of an FASD diagnosis (i.e., presence of 3 neurodevelopmental concerns plus prenatal alcohol exposure), and (b) impairment in each neurodevelopmental domain.

Comprehensive neurodevelopmental assessments can result in the accurate diagnosis of multiple neurodevelopmental disorders. Therefore, a secondary outcome will determine the accuracy of index tests in diagnosing other childhood neurodevelopmental disorders that co-occur, or occur independently of FASD, such as attention-deficit hyperactivity disorder (ADHD) and autism spectrum disorders (ASD).

Study sites and participants

For peer review only - http://bmjopen.bmj.com/site/about/guidelines.xhtml

At least two hundred children referred to one of 8 partner organisations conducting neurodevelopmental assessments will complete index tests and reference tests between April 2022 and April 2025. Inclusion criteria are:

- 6-16 years-old;
- suspected or confirmed prenatal alcohol exposure upon referral;
- able to speak English;

There are no exclusion criteria. Sites include publicly and privately funded clinics [e.g., Aboriginal Community Controlled Health Services (ACCHO), state-funded hospital outpatient clinics, and university-based outpatient clinics]. Site catchment areas cover remote, rural, and metropolitan children. Annually, the proportion of First Nations children who attend sites for a neurodevelopmental assessment range from 20% to 90%.

Sample size

Sites conduct between 1-4 neurodevelopmental assessments per month as part of usual care. The number of FASD diagnoses completed at sites in the previous year was used to project a total recruitment sample of 288 children over three years of data collection. Allowing for 30% attrition, a final sample of 200 participants with 50% representation from rural and remote sites, and 50% representation of First Nations children will be possible.

The probability of FASD diagnosis in this sample will be moderate (estimated at 50-60% across sites) because children are referred to sites due to significant developmental delays and suspected prenatal alcohol exposure. Two hundred participants provide a large enough sample for precise estimates of sensitivity and specificity. For example,

For peer review only - http://bmjopen.bmj.com/site/about/guidelines.xhtml

assuming 55% of the sample have FASD, and the true sensitivity and specificity is 80% and 90% respectively, we will be able to estimate sensitivity to within ±7.5% and specificity to within ±6.2% (alpha=0.05).

Measures

(i) Participant information

Demographic information about the family (family composition, parents' education level and employment status) and about the child (sex, age, date of birth and cultural background) will be collected.

(ii) Clinical assessment information

Child developmental history will be obtained from caregiver interviews and will include: gestational age at birth, prenatal exposures (e.g., prescription medication, illicit drug use), birth complications, family and child medical and mental health history, children's adverse life events, and parent reports of children's learning, speech, behavioural and emotional difficulties. Where possible, *maternal alcohol consumption in pregnancy* will be assessed using the 3-item Alcohol Use Disorders Identification Test-Concise (AUDIT-C).[10] Each item is scored from 0 to 4, with higher scores indicating increased alcohol usage. Total scores range from 0 (no alcohol consumption) to 12; a score of 3 or above for women indicates hazardous drinking. The AUDIT-C has convergent validity with the 10-item version of the scale.[11] *Sentinel Features,* including the standard frontal, three quarter and lateral facial photographs will be collected and analysed using the FAS Facial Photographic Analysis Software 2.0.[12]

For peer review only - http://bmjopen.bmj.com/site/about/guidelines.xhtml

Index Tests were selected by clinical specialists with expertise in FASD, subsequently approved by site practitioners and the project steering committee. Table 1 outlines the rationale for index test selection.

Table 1: Rationale for selection of index tests

Rapid Neurodevelopmental Assessment[13]	Behaviour Assessment System for Children-3[14]
delivered by non-specialists trained to certification in administration, important for rural and remote sites lacking specialists	established reliability and validity within the Australian context[15]
developed for use in low social-economic status populations,[16, 17] important for rural and remote Australians where 13.3% of the population live below the poverty line[18]	covers a broad array of neurodevelopmental domains, including executive and adaptive function, key FASD diagnostic domains not commonly assessed in other parent-report measures of child psychosocial functioning[19]
appropriate for children 0-16 years old, not limited to young children	
utilises independent, direct, observational assessment of neurodevelopment, not solely reliant on parent-report	
categorises impairment according to severity (none, mild, moderate, severe), not dichotomous (e.g., the presence or absence of impairment)	
developed in a non-English speaking country, important for culturally diverse populations and children with English as a second language	

The Rapid Neurodevelopmental Assessment (RNDA) is a 30-minute observational and functional assessment identifying neurodevelopmental impairment in children from birth to 16 years. As well as screening for vision and hearing problems (that can affect task performance), it assesses motor (gross and fine), language, cognition, memory, attention, behaviour, and self-care. A series of age-appropriate structured tasks delivered by trained administrators follow specified decision rules detailed in the

For peer review only - http://bmjopen.bmj.com/site/about/guidelines.xhtml

administration and scoring manual.[13] As a functional assessment (rather than a normed assessment), children are given prompts and modelling to complete tasks if needed. Degree of impairment is classified as: no impairment, mild, moderate, or severe impairment. Research demonstrates that the RNDA has sound to excellent inter-rater reliability across the neurodevelopmental domains with infant, child, and adolescent populations.[20-22] Items have been reviewed by First Nations health practitioners as appropriate for administration with First Nations children. *The Behavioural Assessment Scale for Children, 3rd Edition (BASC-3)* is a 20-minute parent- and teacher-report of 2- to 21-year-old behavioural, emotional and social skills, as well as adaptive, attention and executive functioning in home, school and community settings.[13] Both parent- and teacher-report forms have excellent internal validity, very good test-retest reliability, and inter-rater reliability.[23]

Reference Tests were determined over a 6-month cross-site consultation process. Agreement on reliable and valid measures for 9 domains was achieved in six steps (see Table 2).

Table 2: Steps to establish cross-site agreement for reference tests

Step	Activity
1	An international survey of clinical practice broadly ascertained the standard assessment protocol for each domain at neurodevelopmental assessment clinics globally.
2	A systematic review outlined the standard assessment protocol for each domain when detecting impairment for an FASD diagnosis in internationally published research[24, 25]
3	Identification of tools from Step 1 and 2 with established Australian norms
4	A presentation to lead practitioners at each site introducing outcomes of Step 1, 2, and 3. Lead practitioners took information to their clinical team to compare with site specific assessment protocols for each domain

For peer review only - http://bmjopen.bmj.com/site/about/guidelines.xhtml

5	Monthly group meetings with lead practitioners across sites discussed pros and cons of preferred reference tests, until consensus was achieved.
6	When consensus could not be achieved on domain tests, the underlying construct of the domain was defined, and consensus was achieved on subtests measuring similar constructs. For example, for academic achievement, a valid and reliable reading subtest exists in both the Wide Range Achievement Test[26] and the Wechsler Individual Achievement Test.[27]

Table 3 outlines how the index tests correspond to reference tests selected for each neurodevelopmental domain used to diagnose FASD (excluding brain structure). All reference tests align with recommendations from the Australian Guide to the Diagnosis of FASD.[5]

Table 3: Index and reference tests used to assess neurodevelopmental domains

**Domain**	**Index Test**	**Reference Test**
Motor	RNDA: Gross motor and fine motor subscale	M-ABC 2 or BOT-2
Cognition	RNDA: Cognition subscale	WISC-V
Language	RNDA: Language subscale; BASC-3: Functional communication scale	CELF-5*
Academic Achievement	BASC-3: Learning problems scale	WIAT-III or WRAT-5 or WJ-IV
Memory	RNDA: Memory subscale	CMS: Stories and Dot Location subscales
Attention	RNDA: Behavior subscale (attention items); BASC-3: Attention Problems clinical scale	Connors-3
Executive Function	BASC-3: Executive Functioning Index score	BRIEF-2
Affect Regulation	RNDA: Behavior subscale (affective items); BASC-3: Anxiety and Depression clinical scale	Previous diagnostic status (depression / anxiety)

For peer review only - http://bmjopen.bmj.com/site/about/guidelines.xhtml

Adaptive Behavior, Social Skills, Social Communication	RNDA: Self Care subscale; RNDA: Behavior subscale (peer play item); BASC-3: Adaptive Skills composite	ABAS-3 or Vineland-3

* equivalent language assessment validated for a First Nations population
Note:

- Rapid Neurodevelopmental Assessment: Australian Edition (RNDA)[13]
- Behaviour Assessment System for Children, 3rd Edition (BASC-3)[23]
- Movement Assessment Battery for Children: 2nd Edition (M-ABC 2)[28]
- Bruininks-Oseretsky Test of Motor Proficiency, 2nd Edition (BOT-2)[29]
- Wechsler Intelligence Scale for Children, Version 5 (WISC-V),[30] Australian and New Zealand Standardised Edition
- Clinical Evaluation of Language Fundamentals, Version 5 (CELF-5);[31]
- Wechsler Individual Achievement Test, 3rd Edition (WIAT-III)[27]
- Wide Range Achievement Test, 5th Edition (WRAT-5)[26]
- Woodcock-Johnson Achievement Test, 4th Edition, Australasian Adaptation (WJ-IV)[32]
- Children's Memory Scale (CMS)[33]
- Connors, 3rd Edition[34]
- Behaviour Rating Inventory of Executive Function, 2nd Edition (BRIEF-2)[35]
- Adaptive Behaviour Assessment System, 3rd Edition (ABAS-3)[36]
- Vineland Adaptive Behaviour Scales, 3rd Edition (Vineland-3)[37]

Procedure

Practitioners at each site will ask families with children who meet inclusion criteria if they consent to participate. Each site will administer index and reference tests as clinically appropriate. Index tests will be administered by trained practitioners including Aboriginal Health Workers, nurses, midwives, allied health, GPs and/or paediatricians (when available). Reference tests will be administered by allied health clinicians with relevant qualifications and credentialing for the tool (e.g., psychologists, occupational therapists, speech therapists), who have training and experience diagnosing FASD, consistent with the Australian Guide to the Diagnosis of FASD.[5] Where possible, index tests and reference tests will be administered by different practitioners. Practitioners administering index tests will not have access to reference test results, and vice versa, until all tests are scored. When index or reference tests have been completed by third parties (e.g., the school), families will be asked for their

For peer review only - http://bmjopen.bmj.com/site/about/guidelines.xhtml

consent to share data with participating study sites. A member of the research team will audit health records to extract test scores from completed assessments.

All practitioners and the research team will receive training in study protocols to ensure fidelity prior to administering tests and extracting data. Training will include: purpose of the project, rationale for test selection, and use of the RNDA. RNDA training includes studying standardised training videos developed by certified RNDA trainers, scoring three training tapes, video recording assessments with two sample children, and a review of these videos by a certified scorer (to ensure fidelity). If a site does not have practitioners available to complete a test, clinicians with relevant qualifications from the research team will administer the missing test. Check-in meetings with practitioners at each site will be held quarterly to ensure continued fidelity with study protocols. All index and reference tests aim to be completed within 6-months of the administration of the first test.

Phase II: Economic analysis

Study design

A cost-efficiency analysis will determine the cost and potential economic net benefits of: (a) implementing the rapid index tests compared to comprehensive reference tests, and (b) conducting assessments in primary healthcare compared to specialist services.

Primary and Secondary Outcomes

Economic analyses will be secondary outcomes, which include: the (1) direct healthcare costs, and (2) indirect costs of diagnosing FASD using index tests compared to reference tests; (3) the direct healthcare costs, and (4) indirect cost savings of delivering tests in primary healthcare compared to specialist clinics.

For peer review only - http://bmjopen.bmj.com/site/about/guidelines.xhtml

Participants

Two hundred families enrolled in Phase I will be asked about caregiver costs associated with index tests and reference tests. All sites will record health service costs associated with index tests and reference tests. Sites conducting comprehensive reference FASD assessments prior to the study will record health service costs of usual care for FASD assessments retrospectively.

Measures

(i) *Caregiver costs*

Direct and indirect assessment costs for each family including clinic fees, childcare, accommodation, and meals for assessment days will be surveyed from caregivers.

(ii) *Health service costs*

Prospective cost of delivering *index and reference tests* to participating children will be surveyed from practitioners and include: (1) discipline of the practitioner administering the test; (2) mode of delivery (e.g., clinic, home, school, or telehealth); (3) travel time and costs; (4) test administration time; and (5) indirect administration time for the assessment (e.g., scoring and interpretation).

Usual cost of *reference tests* will be collected from clinic managers in a structured interview, including time taken to administer and score each measure, travel time and other costs.

Procedure

For peer review only

For peer review only - http://bmjopen.bmj.com/site/about/guidelines.xhtml

Prior to participant recruitment, the cost of index and reference tests (usual care) for health services will be retrospectively collected in a structured phone/video interview from site managers. Site manager responses will be recorded, transcribed, and data will be extracted. Expected completion time for index and reference tests will also be extracted from test manuals. Families will be recruited and enrolled in the study as outlined in Phase I. Enrolled families and healthcare practitioners will complete surveys that capture prospective cost information at the completion of testing. Therefore, the cost of index and reference tests will be collected retrospectively for tests administered as part of prior usual care from health service interviews, and costs will be collected prospectively for patients and health services recruited during the study period.

Patient and public involvement

The rapid assessment used in the current project forms part of a tiered approach to screening, diagnosing and supporting FASD in remote Australian communities that was co-designed with First Nations Elders. Co-design involved First Nations Elders, a community advisory group, First Nations remote health practitioners, clinical experts and researchers. The research questions in the current project originated from community advisory group because they asked, 'What is necessary and sufficient to screen for and diagnose FASD?' (ensuring judicious investment of resources), and 'Who in the health care system is best placed to diagnose FASD in a remote setting?' (reducing bottlenecks in specialist settings). Ongoing collaboration (focus groups, informal yarning, 3-monthly 1-week visits to community) between this group and the research team supported the development of the current protocol and this group has agreed to continue support for the duration of the project. Practitioners at new partner sites will be asked to identify a community representative interested in joining our

For peer review only - http://bmjopen.bmj.com/site/about/guidelines.xhtml

advisory group. The advisory group will be informed of project progress and at the end of data collection, they will be involved in interpreting study results and will support dissemination of findings to local communities and the general public.

Data analysis

Phase I: Diagnostic accuracy

All data will be entered into a REDCap database hosted by [redacted for blind review].[38] Data will be identifiable until all test results and survey data are matched to participants. Data will be de-identified for analysis. Pre-specified test positivity cutoffs reflecting clinical norms (e.g., T score>70) will be used, receiver operating curves will also be used to explore best possible cutoffs. Standard diagnostic statistics will be calculated, including: receiver operating curves, sensitivity, specificity, positive and negative predictive values, and likelihood ratios (using 95% confidence intervals).[39] Kappa statistics will be used to compare the reliability of the classification of children as normal/moderately impaired vs. severe on each measure of the neurodevelopmental domains.[40] Continuous scores are available for many of the assessments, which will be used to calculate correlations between matched scores from the index tests and reference tests. Bland-Altman plots will examine the agreement between matched index test and reference test scores, overall and by domain.[40] These plots will also help to determine if any difference between the index and the reference is influenced by the actual scores on the tests (e.g., if the index-standard difference is larger when children have low or high scores). Diagnostic outcomes from the index tests will be compared to diagnostic outcomes from the reference tests to explore whether the index tests function as an effective screening tool for FASD.

For peer review only - http://bmjopen.bmj.com/site/about/guidelines.xhtml

Phase II: Economic analysis

A multivariable generalised linear model of the cost of diagnosis, with the predictive variable as the index test or reference test, will estimate the incremental cost between the two alternative diagnostic processes adjusting for differences in patient geographical location and other potential confounders. Sub-group cost analysis will explore heterogeneity of results between patients' subgroups by including an interaction term (e.g., location, diagnostic procedure) within the multivariable generalised linear model of cost. The expected value of implementation will be calculated as the net monetary benefit of the index tests (i.e., monetary benefits – costs) multiplied by the population of patients expected to benefit from the reference tests. Probabilistic and one way sensitivity analysis will be used to characterise the uncertainty in the economic results, highlighting the most important drivers for the value of implementing the index tests.

Summary

This research aims to establish the accuracy and cost-efficiency of rapid, culturally-responsive screening and assessment tools, which are part of a co-designed, tiered neurodevelopmental assessment process for diagnosing FASD in primary healthcare.[41-44] Diagnosing children with FASD in primary healthcare will lessen the burden on specialist systems and help to place developmentally and socially/emotionally vulnerable children on support trajectories earlier. Our findings will help to demonstrate how primary healthcare, which is readily available to families regardless of their location, can support the assessment of FASD. Even partially adding information available to specialists can streamline subsequent interventions. Providing primary

For peer review only - http://bmjopen.bmj.com/site/about/guidelines.xhtml

healthcare practitioners with the knowledge and tools to quickly identify children who are developmentally at-risk, to understand when to diagnose children, and when to seek specialist involvement, will reduce the demand on over-burdened specialist clinics. This will assist in providing timely access for assessment and treatment of children suspected of having FASD in Australia.

ETHICS AND DISSEMINATION

Ethics

The study has received ethical approval from the [redacted for blind peer review] Human Research Ethics Committee (2020/743) and [redacted for blind peer review] Human Research Ethics Committee (HREC/20/XXXX/63173). Consent to conduct research was obtained from the [redacted for blind peer review], representing the Traditional Owners of the lands from remote data collection sites.

Dissemination of findings

All study data will be archived on a secure server at [redacted for blind peer review] for at least five years after the last publication. Re-identifiable participant data will be collected on a secure web-based platform (REDCap) and exported to [redacted for blind peer review] server for archiving. A summary report will be disseminated to participating site practitioners and partner organisations. The community advisory group will support dissemination of outcomes within local communities. Outcomes of the project will be disseminated widely at national and international conferences, published in peer-reviewed journals and disseminated on social media.

Author contributions

For peer review only - http://bmjopen.bmj.com/site/about/guidelines.xhtml

DCS is the principal Chief Investigator, conceptualised the project, supervised the design and implementation of the research, and wrote the first draft of the protocol. EH conceptualised the project, planned the assessment protocols, data collection and analyses, and revised the protocol. MJZ-G and AJW participated in the project conceptualisation and design of the protocol, advise through membership on the steering committee, and contributed to preparation of the manuscript. JB and RSW advised on methodological and analytical aspects of the project, coordinate the health economics data collection and analyses, and critically revised the protocol. GS contributed to writing the first draft of the protocol and incorporating co-author revisions. WL contributed to project conceptualisation, manages project implementation, and revised the protocol. CW helped plan data collection, manages project data and storages procedures, and contributed to writing the protocol. SH, MP, DS, HT, IM, CH, KH, and AC are partner investigators on this project. They coordinate site project implementation, collects data, participate in monthly steering committee meetings and revised the manuscript. NR contributed to conceptualisation of this project, coordinates site data collection, and revised the manuscript. KM, SR, KW and SR-T are chief investigators who participate in steering committee meetings and revised the manuscript.

Acknowledgements

We would like to acknowledge the many contributors to the DATAforFASD project. This includes the participating families, who graciously contribute extra time to complete lengthy assessments so that those in the future might get faster access to care. We thank the healthcare practitioners, who have added to their workload by incorporating this protocol into their existing clinical assessments. We thank the partner

For peer review only - http://bmjopen.bmj.com/site/about/guidelines.xhtml

investigators, chief investigators, associate investigators and steering committee who have guided the conceptualisation and implementation of this project. We would like to thank our Community Advisory Group who started the journey to think differently about how to assess FASD so that more children could receive care close to home. We would like to thank [redacted for blind review]. The project would not have been possible without their gracious support.

Funding statement

This work was funded by an Australian National Health and Medical Research Council Partnership Grant [redacted for blind review].

Patient consent for publication

Not required.

Competing interests

The authors report that they have no competing interests, financial or otherwise.

Registration details for study

Trial Registration Number**:** ACTRN12622000498796

For peer review only - http://bmjopen.bmj.com/site/about/guidelines.xhtml

REFERENCES

1. Lange S, Probst C, Gmel G, *et al.* Global prevalence of fetal alcohol spectrum disorder among children and youth: a systematic review and meta-analysis. *JAMA Pediatr*. 2017;171:948-56.
2. May PA, Chambers CD, Kalberg WO, *et al.* Prevalence of fetal alcohol spectrum disorders in 4 US communities. *JAMA*. 2018;319:474-82.
3. Fitzpatrick JP, Latimer J, Carter M, *et al.* Prevalence of fetal alcohol syndrome in a population-based sample of children living in remote Australia: the Lililwan project. *J Paediatr Child Health*. 2015;51:450-7.
4. Popova S, Lange S, Probst C, *et al.* Estimation of national, regional, and global prevalence of alcohol use during pregnancy and fetal alcohol syndrome: a systematic review and meta-analysis. *The Lancet Global Health*. 2017;5:e290-e9.
5. Bower C, Elliott EJ, Zimmet M, *et al.* Australian guide to the diagnosis of foetal alcohol spectrum disorder: a summary. *J Paediatr Child Health*. 2017;53:1021.
6. McCleary K. Utilising a simulation-based teaching strategy to develop a new physiotherapy paediatric critical care service in a developing tertiary health service. *Aust Criti Care*. 2020;33:S17.
7. The Lowitja Institute. Close the gap: We nuture our culture for our future, and our culture nurtures us. In: Justice AaTSIS, editor. Australia: The Close the Gap Campaign Steering Committee; 2020.
8. Reid N, White C, Hawkins E, *et al.* Outcomes and needs of health and education professionals following fetal alcohol spectrum disorder-specific training. *J Paediatr Child Health*. 2020;56:317-23.
9. Bossuyt PM, Reitsma JB, Bruns DE, *et al.* STARD 2015: an updated list of essential items for reporting diagnostic accuracy studies. *Clinical Chem*. 2015;61:1446-52.
10. Bush K KD, McDonell MB, *et al.* The audit alcohol consumption questions (AUDIT-C): an effective brief screening test for problem drinking. *Arch Intern Med*. 1988;158:1789-95.
11. Bradley KA, Bush KR, Epler AJ, *et al.* Two brief alcohol-screening tests from the alcohol use disorders identification test (AUDIT-C): validation in a female veterans affairs patient population. *Arch Intern Med*. 2003;163:821-9.
12. Hemingway SJA, Bledsoe JM, Brooks A, *et al.* Comparison of the 4-digit code, Canadian 2015, Australian 2016 and Hoyme 2016 fetal alcohol spectrum disorder diagnostic guidelines. *Adv in Pediatric Resear*. 2019;6.
13. Khan N, Muslima H. Rapid neurodevelopmental assessment of children: a practical guide for multidisciplinary professionals. Dhaka: Bangladesh Protibondhi Foundation; 2017.
14. Reynolds C, Kamphaus R. Behaviour assessment system for children – third edition manual: Circle Pines, MN: American Guidance Service; 2015.
15. Anggun T. S. T., Kraska J, Bell K., *et al.* Confirmatory factor analyses of the behavior assessment system for children – third edition among an australian sample. *Edu & Dev Psych*. 2021;38:131-42.
16. Muslima H, Khan NZ, Shilpi AB, *et al.* Validation of a rapid neurodevelopmental assessment tool for 10- to 16-year-old young adolescents in Bangladesh. *Child: Care, Health and Dev*. 2016;42:658-65.
17. Thompson L, Peñaloza RA, Stormfields K, *et al.* Validation and adaptation of rapid neurodevelopmental assessment instrument for infants in Guatemala. *Child: Care, Health and Dev*. 2015;41:1131-9.

For peer review only

18. National Rural Health Alliance I. Mental health in rural and remote Australia: Fact sheet. 2017.
19. Flannigan KR, Coons-Harding KD, Turner O, *et al.* A survey of measures used to assess brain function at fasd clinics in Canada. *Canadian Psych*. 2022;63:106-19.
20. Khan NZ, Muslima H, Begum D, *et al.* Validation of rapid neurodevelopmental assessment instrument for under-two-year-old children in Bangladesh. *Pediatr*. 2010;125:e755-e62.
21. Khan NZ, Muslima H, Shilpi AB, *et al.* Validation of rapid neurodevelopmental assessment for 2-to 5-year-old children in Bangladesh. *Pediatr*. 2013;131:e486-e94.
22. Khan NZ, Muslima H, El Arifeen S, *et al.* Validation of a rapid neurodevelopmental assessment tool for 5 to 9 year-old children in Bangladesh. *Pediatr*. 2014;164:1165-70. e6.
23. Kamphaus RW, Reynolds CR. Behavioral and emotional screening system (BASC): Manual. Pearson Inc.; 2007.
24. Reid N, Shanley DC, Logan J, *et al.* International survey of specialist fetal alcohol spectrum disorder diagnostic clinics: comparison of diagnostic approach and considerations regarding the potential for unification. *Inter J Environ Resear and Public Health*. 2022;19:15663.
25. Reid N, Hawkins E, White C, *et al.* Is a unified approach to fasd diagnosis possible? Results of an international surveyof diagnostic clinics. In: Alcoholism-clinical experimental research. NJ, USA: Willey; 2021.
26. Wilkinson GS, J. RG. Wide range achievement test, fifth edition (WRAT-5). 2017.
27. Wechsler D. Wechsler individual achievement test (3rd ed.). San Antonio, TX: Pearson; 2009.
28. Henderson SE, Sugden D, Barnett AL. Movement assessment battery for children-2. *Resear in Dev Disabilities*. 2007.
29. Bruininks RH. Bruininks-oseretsky test of motor proficiency: BOT-2: NCS Pearson/AGS Minneapolis, MN; 2005.
30. Terriot K, Ozenne R. Wechsler intelligence scale for children, v version (WISC-v). ANAE - Approche Neuropsychologique des Apprentissages chez l'Enfant. 2015;27:95-102.
31. Wiig EH, Semel E, Secord WA. Clinical evaluation of language fundamentals-fifth edition (CELF-5). Bloomington, MN: NCS Pearson; 2013.
32. Schrank FA, McGrew KS, Mather N. Woodcock johnson fourth edition - australasian adaptation Rolling Meadows, IL: Riverside; 2014.
33. Cohen MJ. Children’s memory scale. In: Kreutzer JS, DeLuca J, Caplan B, editors. Encyclopedia of clinical neuropsychology. New York, NY: Springer New York; 2011. p. 556-9.
34. Conners CK, Pitkanen J, Rzepa SR. Conners 3rd edition. In: Kreutzer JS, DeLuca J, Caplan B, editors. Encyclopedia of clinical neuropsychology. New York, NY: Springer New York; 2011. p. 675-8.
35. Gioia GA, Isquith PK, Guy SC, *et al.* Test review behavior rating inventory of executive function. *Child Neuropsy*. 2000;6:235-8.
36. Harrison PL, Oakland T. Adaptive behavior assessment system: Psychological Corporation San Antonio, TX; 2000.
37. Sparrow SS, Cicchetti DV, Saulnier CA. Vineland adaptive behavior scales, third edition. Bloomington, MN: NCS Pearson, Inc.; 2016.

For peer review only

38. Patridge EF, Bardyn TP. Research electronic data capture (REDCap). *J Med Library Asso: JMLA*. 2018;106:142.
39. Chrobak K. Studying a study & testing a test: Reading evidence-based health research 6th ed. *J Canadian Chiro Asso*. 2013;57:96-.
40. Morgan CJ, Aban I. Methods for evaluating the agreement between diagnostic tests. Springer; 2016.
41. Shanley DC, Hawkins E, Page M, *et al.* Protocol for the yapatjarrathati project: a mixed-method implementation trial of a tiered assessment process for identifying fetal alcohol spectrum disorders in a remote australian community. *BMC Health Services Research*. 2019;19:1-11.
42. Reid N, Page M, McDonald T, *et al.* Integrating cultural considerations and developmental screening into an australian first nations child health check. *Aust J Primary Health*. 2022.
43. Reid N, Hawkins E, Liu W, *et al.* Yarning about fetal alcohol spectrum disorder: outcomes of a community-based workshop. *Resear in Dev Disabilities*. 2021;108:103810.
44. Miller L, Shanley DC, Page M, *et al.* Preventing drift through continual co-design: refining the prototype of a tiered fasd assessment. *Inter J Environ Resear and Public Health*. 2022;18.

For peer review only

Section & Topic	No	Item	Reported on page #
**TITLE OR ABSTRACT**			
	**1**	Identification as a study of diagnostic accuracy using at least one measure of accuracy (such as sensitivity, specificity, predictive values, or AUC)	1
**ABSTRACT**			
	**2**	Structured summary of study design, methods, results, and conclusions (for specific guidance, see STARD for Abstracts)	2-3
**INTRODUCTION**			
	**3**	Scientific and clinical background, including the intended use and clinical role of the index test	6
	**4**	Study objectives and hypotheses	6
**METHODS**			
Study design	**5**	Whether data collection was planned before the index test and reference standard were performed (prospective study) or after (retrospective study)	7
Participants	**6**	Eligibility criteria	8
	**7**	On what basis potentially eligible participants were identified (such as symptoms, results from previous tests, inclusion in registry)	8
	**8**	Where and when potentially eligible participants were identified (setting, location and dates)	8
	**9**	Whether participants formed a consecutive, random or convenience series	8
Test methods	**10a**	Index test, in sufficient detail to allow replication	10-11
	**10b**	Reference standard, in sufficient detail to allow replication	11-12
	**11**	Rationale for choosing the reference standard (if alternatives exist)	12-13
	**12a**	Definition of and rationale for test positivity cut-offs or result categories of the index test, distinguishing pre-specified from exploratory	17
	**12b**	Definition of and rationale for test positivity cut-offs or result categories of the reference standard, distinguishing pre-specified from exploratory	17
	**13a**	Whether clinical information and reference standard results were available to the performers/readers of the index test	13
	**13b**	Whether clinical information and index test results were available to the assessors of the reference standard	13
Analysis	**14**	Methods for estimating or comparing measures of diagnostic accuracy	17
	**15**	How indeterminate index test or reference standard results were handled	Protocol (no data)
	**16**	How missing data on the index test and reference standard were handled	Protocol (no data)
	**17**	Any analyses of variability in diagnostic accuracy, distinguishing pre-specified from exploratory	Protocol (no data)
	**18**	Intended sample size and how it was determined	8-9
**RESULTS**			
Participants	**19**	Flow of participants, using a diagram	Protocol (no data)
	**20**	Baseline demographic and clinical characteristics of participants	9 (planned)
	**21a**	Distribution of severity of disease in those with the target condition	Protocol (no data)
	**21b**	Distribution of alternative diagnoses in those without the target condition	Protocol (no data)
	**22**	Time interval and any clinical interventions between index test and reference standard	14
Test results	**23**	Cross tabulation of the index test results (or their distribution) by the results of the reference standard	17
	**24**	Estimates of diagnostic accuracy and their precision (such as 95% confidence intervals)	17
	**25**	Any adverse events from performing the index test or the reference standard	19
**DISCUSSION**			
	**26**	Study limitations, including sources of potential bias, statistical uncertainty, and generalisability	18
	**27**	Implications for practice, including the intended use and clinical role of the index test	18
**OTHER INFORMATION**			
	**28**	Registration number and name of registry	21

For peer review only - http://bmjopen.bmj.com/site/about/guidelines.xhtml

CC BY SA

	**29**	Where the full study protocol can be accessed	This is the full study protocol
	**30**	Sources of funding and other support; role of funders	20

For peer review only

STARD 2015

AIM

STARD stands for “Standards for Reporting Diagnostic accuracy studies”. This list of items was developed to contribute to the completeness and transparency of reporting of diagnostic accuracy studies. Authors can use the list to write informative study reports. Editors and peer-reviewers can use it to evaluate whether the information has been included in manuscripts submitted for publication.

EXPLANATION

A **diagnostic accuracy study** evaluates the ability of one or more medical tests to correctly classify study participants as having a **target condition.** This can be a disease, a disease stage, response or benefit from therapy, or an event or condition in the future. A medical test can be an imaging procedure, a laboratory test, elements from history and physical examination, a combination of these, or any other method for collecting information about the current health status of a patient.

The test whose accuracy is evaluated is called **index test.** A study can evaluate the accuracy of one or more index tests. Evaluating the ability of a medical test to correctly classify patients is typically done by comparing the distribution of the index test results with those of the **reference standard**. The reference standard is the best available method for establishing the presence or absence of the target condition. An accuracy study can rely on one or more reference standards.

If test results are categorized as either positive or negative, the cross tabulation of the index test results against those of the reference standard can be used to estimate the **sensitivity** of the index test (the proportion of participants *with* the target condition who have a positive index test), and its **specificity** (the proportion *without* the target condition who have a negative index test). From this cross tabulation (sometimes referred to as the contingency or “2x2” table), several other accuracy statistics can be estimated, such as the positive and negative **predictive values** of the test. Confidence intervals around estimates of accuracy can then be calculated to quantify the statistical **precision** of the measurements.

If the index test results can take more than two values, categorization of test results as positive or negative requires a **test positivity cut-off**. When multiple such cut-offs can be defined, authors can report a receiver operating characteristic (ROC) curve which graphically represents the combination of sensitivity and specificity for each possible test positivity cut-off. The **area under the ROC curve** informs in a single numerical value about the overall diagnostic accuracy of the index test.

The **intended use** of a medical test can be diagnosis, screening, staging, monitoring, surveillance, prediction or prognosis. The **clinical role** of a test explains its position relative to existing tests in the clinical pathway. A replacement test, for example, replaces an existing test. A triage test is used before an existing test; an add-on test is used after an existing test.

Besides diagnostic accuracy, several other outcomes and statistics may be relevant in the evaluation of medical tests. Medical tests can also be used to classify patients for purposes other than diagnosis, such as staging or prognosis. The STARD list was not explicitly developed for these other outcomes, statistics, and study types, although most STARD items would still apply.

DEVELOPMENT

This STARD list was released in 2015. The 30 items were identified by an international expert group of methodologists, researchers, and editors. The guiding principle in the development of STARD was to select items that, when reported, would help readers to judge the potential for bias in the study, to appraise the applicability of the study findings and the validity of conclusions and recommendations. The list represents an update of the first version, which was published in 2003.

More information can be found on http://www.equator-network.org/reporting-guidelines/stard.

For peer review only - http://bmjopen.bmj.com/site/about/guidelines.xhtml

BMJ Open

Diagnostic Accuracy and economic value of a Tiered Assessment for Fetal Alcohol Spectrum Disorder (DATAforFASD): Protocol

Journal:	*BMJ Open*
Manuscript ID	bmjopen-2022-071004.R1
Article Type:	Protocol
Date Submitted by the Author:	28-Jun-2023
Complete List of Authors:	Shanley, Dianne; Griffith University, Menzies Health Institute of Queensland/School of Applied Psychology<br>Zimmer-Gembeck, Melanie ; Griffith University, Menzies Health Institute of Queensland/School of Applied Psychology<br>Wheeler, Amanda; Griffith University, Menzies Health Institute of Queensland; The University of Auckland, Faculty of Medical & Health Sciences<br>Byrnes, Joshua; Griffith University, Menzies Health Institute of Queensland/School of Medicine and Dentistry<br>Ware, Robert; Griffith University, Menzies Health Institute of Queensland<br>Liu, Wei; Griffith University, Menzies Health Institute of Queensland<br>Simcock, Gabrielle; Griffith University, Menzies Health Institute of Queensland<br>White, Codi; Griffith University, Menzies Health Institute of Queensland<br>Horton, Sarah; Gidgee Healing, Family Health; Griffith University, Menzies Health Institute of Queensland<br>Page, Marjad; Kambu Aboriginal and Torres Strait Islander Corporation for Health<br>Shelton, Doug; Gold Coast Hospital and Health Service; Griffith University, Menzies Health Institute of Queensland/School of Medicine and Dentistry<br>Till, Haydn; Gold Coast Hospital and Health Service; Griffith University, Menzies Health Institute of Queensland/School of Medicine and Dentistry<br>Mills, Ianthe; Sunshine Coast Hospital and Health Service<br>Hislop, Carly; Townsville Hospital and Health Service, Child Development Service<br>Harris, Katrina ; Monash Health, Victorian Fetal Alcohol Service; Monash University, Department of Paediatrics<br>Crichton, Alison; Monash Health, Victorian Fetal Alcohol Service; Monash University, Department of Paediatrics<br>Reid, Natasha; The University of Queensland,<br>Reilly, Sheena; Griffith University,<br>Moritz, Karen; The University of Queensland, ; The University of Queensland Child Health Research Centre<br>Walsh, K.; Queensland University of Technology,<br>Rundle-Thiele, Sharyn; Griffith University,<br>Hawkins, Erinn; Griffith University, School of Applied Psychology/Menzies Health Institute of Queensland

**Primary Subject Heading**:	Diagnostics
Secondary Subject Heading:	Paediatrics
Keywords:	PRIMARY CARE, HEALTH ECONOMICS, Community child health < PAEDIATRICS, Developmental neurology & neurodisability < PAEDIATRICS, Health Services Accessibility

Title: Diagnostic Accuracy and economic value of a Tiered Assessment for Fetal Alcohol Spectrum Disorder (DATAforFASD): Protocol

Running Head: DATAforFASD Protocol

Dianne C. Shanley[a,b]*, Melanie J. Zimmer-Gembeck[a,b], Amanda J Wheeler[a,c], Joshua Byrnes[a,d], Robert S Ware[a], Wei Liu[a], Gabrielle Simcock[a], Codi White[a], Sarah Horton[g], Marjad Page[a,h], Doug Shelton[a,d,e], Haydn Till[a,d,e], Ianthe Mills[f], Carly Hislop[i], Katrina Harris[j,k], Ali Crichton[j,k], Natasha Reid[l], Sheena Reilly[a], Karen M. Moritz[m], Kerryann Walsh[n], Sharyn Rundle-Thiele[o], and Erinn Hawkins[a,b]

[a] Menzies Health Institute of Queensland, Griffith University, Australia
[b] School of Applied Psychology, Griffith University, Australia
c Faculty of Medical & Health Sciences, University of Auckland, NZ
d School of Medicine and Dentistry, Griffith University, Australia
e Community Child Health, Gold Coast Hospital and Health Service, Australia
f Child and Adolescent Unit, Sunshine Coast Hospital and Health Service, Australia
g Family Health, Gidgee Healing, Australia
h Executive team, Kambu Aboriginal and Torres Strait Islander Corporation for Health, Australia
i Child Development Service, Townsville Hospital and Health Service, Australia
j Victorian Fetal Alcohol Service, Monash Children's Hospital, Australia
k Department of Paediatrics, Monash University, Australia
l Child Health Research Centre, University of Queensland, Australia
m Faculty of Medicine, University of Queensland, Australia
n School of Early Childhood and Inclusive Education, Queensland University of Technology, Australia
o Social Marketing, Griffith University, Australia

***Corresponding author**

Dianne C. Shanley
Menzies Health Institute Queensland, School of Applied Psychology
Griffith University, Gold Coast Campus
Parklands Drive, QLD 4222
Australia
d.shanley@griffith.edu.au

Word count: 3,945

For peer review only

ABSTRACT

Introduction: Australian practices for diagnosing fetal alcohol spectrum disorder (FASD) are lengthy and require specialist expertise. Specialist teams are based in urban locations; they are expensive and have prolonged waitlists. Innovative, flexible solutions are needed to ensure First Nations children living in rural/remote communities have culturally appropriate and equitable access to timely diagnosis and support. This study compares the accuracy of rapid assessments (index tests) that can be administered by a range of primary healthcare practitioners to specialist standardised FASD assessments (reference tests). The cost-efficiency of index tests will be compared to reference tests.

Methods and analysis: At least 200 children aged 6-16 years at-risk of FASD will be recruited across 7 study sites. Following STARD diagnostic accuracy guidelines, all children will complete index and reference tests. Diagnostic accuracy statistics (including receiver operating curves, sensitivity, specificity, positive and negative predictive values, and likelihood ratios) will identify whether rapid assessments can accurately identify: (a) the presence of a FASD diagnosis and (b) impairment in each neurodevelopmental domain, compared to comprehensive assessments. Direct and indirect healthcare costs for index tests compared to reference tests will be collected in primary healthcare and specialist settings.

Ethics and dissemination of results: Children's Health Queensland Hospital and Health Service Human Research Ethics Committee (HREC/20/QCHQ/63173); Griffith University Human Research Ethics Committee (2020/743). Results will assist in validating the use of index tests as part of a tiered neurodevelopmental assessment process that was co-designed with First Nations community and primary healthcare

For peer review only - http://bmjopen.bmj.com/site/about/guidelines.xhtml

practitioners. Outcomes will be summarised and provided to participating practitioners and sites, and disseminated to community health services and consumers. Findings will be presented at national and international conferences and published in peer-reviewed journals.

Registration details: ACTRN12622000498796 (29 March 2022)

Key words: fetal alcohol spectrum disorder; primary care; Indigenous Health; neurodevelopmental disorder; diagnostic tests and procedures; cost-benefit analysis; co-design

For peer review only

For peer review only - http://bmjopen.bmj.com/site/about/guidelines.xhtml

STRENGTHS AND LIMITATIONS OF THIS STUDY

- DATAforFASD is the first large, prospective, multi-site trial to compare comprehensive assessments to rapid assessments across nine domains of neurodevelopment so that we can better understand the accuracy of using rapid assessments to screen and diagnose neurodevelopmental disorders like FASD.
- A sample size of at least 200 children is uncommon in FASD research and may prove to be challenging, because completing gold-standard comprehensive assessments on 9 neurodevelopmental domains requires around 2 days of multi-disciplinary specialist (paediatrician/allied health) time per child.
- The rapid assessment fits within a co-designed tiered assessment process that is responsive to cultural protocols and limited health care resources in remote regions. It includes the Rapid Neuro-developmental Assessment, an observational functional neurodevelopmental assessment, and the Behavioural Assessment Scale for Children, 3rd Ed.
- This project follows STARD guidelines, providing the methodological rigor necessary to assess the diagnostic accuracy of rapid assessments in identifying FASD, as well as their accuracy in identifying neurodevelopmental impairment across 9 domains; the cost benefit of using rapid assessment will be explored for families and health services.
- Establishing valid, accurate rapid assessments will enable: (a) early detection of at-risk children, particularly in rural and remote communities where access to care is inequitable; (b) non-specialist care providers (health, education, youth justice, child protection) to confidently contribute to information needed for diagnostic purposes; (c) enhanced triage, resulting in better quality referrals to specialists; (d) the use of rapid assessments for diagnostic purposes, potentially addressing a systemic

For peer review only - http://bmjopen.bmj.com/site/about/guidelines.xhtml

healthcare challenge by offsetting the longest most costly assessment process for the most prevalent preventable neurodevelopmental disorder.

For peer review only

For peer review only - http://bmjopen.bmj.com/site/about/guidelines.xhtml

INTRODUCTION

Fetal alcohol spectrum disorder (FASD) affects between 1-5% of Australian children, and up to 19% in some remote Australian communities, which is higher than any other neurodevelopmental disorder.[1-4] To identify FASD, current Australian guidelines recommend comprehensive diagnostic assessments (reference tests) across ten neurodevelopmental domains, delivered by multi-disciplinary teams. These assessments are primarily available in Australia at specialist services, making this a lengthier and more costly diagnostic process than any other neurodevelopmental disorder.[5] Current waitlists for Australian children suspected of FASD are up to 3 years at some health services. Overall, specialist services are only seeing a small proportion of suspected cases and children from rural and remote regions are left behind.[6, 7]

To meet the demand for diagnosis, our Australian health system either needs an inordinate number of specialists and multidisciplinary clinics, or we need to think differently about how to diagnose FASD. Our health system is unlikely to be able to resource the longest, most costly assessment process for the most prevalent neurodevelopmental disorder in children, but the use of valid and reliable rapid assessment tools could leverage capacity within primary care, increasing vulnerable children's access to diagnosis.

In this project we will conduct a two-phase study to: (i) determine the diagnostic accuracy of rapid neurodevelopment assessments (index tests) that can be used to detect FASD in primary healthcare (Phase I);[8] and (ii) examine the economic benefits of leveraging primary healthcare to support specialist practitioners in the diagnosis of FASD, in terms of cost for families and healthcare systems (Phase II).

For peer review only

METHODS AND ANALYSIS

Phase I: Diagnostic accuracy

Study design

A prospective diagnostic accuracy study (following STARD guidelines) will compare index tests to reference tests for 9 of 10 neurodevelopmental domains commonly used by specialist, multi-disciplinary teams when diagnosing FASD.[9] The reference test for the brain structure domain is already rapid (i.e., history of seizures, head circumference or brain imaging if available). Therefore, an index test for this domain will not be administered. Information for this domain will still be collected for diagnostic purposes.

Primary and Secondary Outcomes

The first primary outcome will determine the diagnostic accuracy of identifying FASD with index tests compared to reference tests. The second primary outcome will determine the accuracy of identifying impairment within each domain assessed. It is hypothesised that the rapid assessments (index tests) will have good sensitivity and specificity when identifying: (a) the presence of an FASD diagnosis (i.e., presence of 3 neurodevelopmental concerns plus prenatal alcohol exposure), and (b) impairment in each neurodevelopmental domain.

Comprehensive neurodevelopmental assessments can result in the accurate diagnosis of multiple neurodevelopmental disorders. Therefore, a secondary outcome will determine the accuracy of index tests in diagnosing other childhood neurodevelopmental disorders that co-occur, or occur independently of FASD, such as attention-deficit hyperactivity disorder (ADHD) and autism spectrum disorders (ASD).

For peer review only - http://bmjopen.bmj.com/site/about/guidelines.xhtml

Study sites and participants

At least two hundred children referred to one of 7 partner organisations conducting neurodevelopmental assessments will complete index tests and reference tests between April 2022 and April 2025. Sites include the Gold Coast Hospital and Health Service, Sunshine Coast Hospital and Health Service, North West Hospital and Health Service, Townsville Hospital and Health Service, Monash Children's Hospital, Gidgee Healing, and Kambu Aboriginal and Torres Strait Islander Corporation for Health. Additional sites may commence to ensure the projected sample size is reached. Inclusion criteria are:

- 6-16 years-old;
- suspected or confirmed prenatal alcohol exposure upon referral;
- able to speak English;

There are no exclusion criteria. Sites include publicly and privately funded clinics [e.g., Aboriginal Community Controlled Health Services (ACCHO), state-funded hospital outpatient clinics, and university-based outpatient clinics]. Site catchment areas cover remote, rural, and metropolitan children. Annually, the proportion of First Nations children who attend sites for a neurodevelopmental assessment range from 20% to 90%.

Sample size

Sites conduct between 1-4 neurodevelopmental assessments per month as part of usual care. The number of FASD diagnoses completed at sites in the previous year was used to project a total recruitment sample of 288 children over three years of data collection. Allowing for 30% attrition, a final sample of 200 participants with 50% representation

For peer review only - http://bmjopen.bmj.com/site/about/guidelines.xhtml

from rural and remote sites, and 50% representation of First Nations children will be possible.

The probability of FASD diagnosis in this sample will be moderate (estimated at 50-60% across sites) because children are referred to sites due to significant developmental delays and suspected prenatal alcohol exposure. Two hundred participants provide a large enough sample for precise estimates of sensitivity and specificity. For example, assuming 55% of the sample have FASD, and the true sensitivity and specificity is 80% and 90% respectively, we will be able to estimate sensitivity to within ±7.5% and specificity to within ±6.2% (alpha=0.05).

Measures

(i) Participant information

Demographic information about the family (family composition, parents' education level and employment status) and about the child (sex, age, date of birth and cultural background) will be collected.

(ii) Clinical assessment information

Child developmental history will be obtained from caregiver interviews and will include: gestational age at birth, prenatal exposures (e.g., prescription medication, illicit drug use), birth complications, family and child medical and mental health history, children's adverse life events, and parent reports of children's learning, speech, behavioural and emotional difficulties. Where possible, *maternal alcohol consumption in pregnancy* will be assessed using the 3-item Alcohol Use Disorders Identification Test-Concise (AUDIT-C).[10] Each item is scored from 0 to 4, with higher scores

For peer review only - http://bmjopen.bmj.com/site/about/guidelines.xhtml

indicating increased alcohol usage. Total scores range from 0 (no alcohol consumption) to 12; a score of 3 or above for women indicates hazardous drinking. The AUDIT-C has convergent validity with the 10-item version of the scale.[11] *Sentinel Features,* including the standard frontal, three quarter and lateral facial photographs will be collected and analysed using the FAS Facial Photographic Analysis Software 2.0.[12]

Index Tests were selected by clinical specialists with expertise in FASD, subsequently approved by site practitioners and the project steering committee. Table 1 outlines the rationale for index test selection.

Table 1: Rationale for selection of index tests

Rapid Neurodevelopmental Assessment[13]	Behaviour Assessment System for Children-3[14]
delivered by non-specialists trained to certification in administration, important for rural and remote sites lacking specialists	established reliability and validity within the Australian context[15]
developed for use in low social-economic status populations,[16, 17] important for rural and remote Australians where 13.3% of the population live below the poverty line[18]	covers a broad array of neurodevelopmental domains, including executive and adaptive function, key FASD diagnostic domains not commonly assessed in other parent-report measures of child psychosocial functioning[19]
appropriate for children 0-17 years old, not limited to young children	
utilises independent, direct, observational assessment of neurodevelopment, not solely reliant on parent-report	
categorises impairment according to severity (none, mild, moderate, severe), not dichotomous (e.g., the presence or absence of impairment)	
instructions can be non-verbal (shown not spoken), , important for culturally diverse populations and children with English as a second language	

For peer review only - http://bmjopen.bmj.com/site/about/guidelines.xhtml

The Rapid Neurodevelopmental Assessment (RNDA) is a 30-minute observational and functional assessment identifying neurodevelopmental impairment in children from birth to 16 years. As well as screening for vision and hearing problems (that can affect task performance), it assesses motor (gross and fine), language, cognition, memory, attention, behaviour, and self-care. A series of age-appropriate structured tasks delivered by trained administrators follow specified decision rules detailed in the administration and scoring manual.[13] As a functional assessment (rather than a normed assessment), children are given prompts and modelling to complete tasks if needed. Degree of impairment is classified as: typical, mild, moderate, or severe impairment. Research demonstrates that the RNDA has sound to excellent inter-rater reliability across the neurodevelopmental domains with infant, child, and adolescent populations.[20-22] The RNDA has been reviewed by First Nations health practitioners and r administered with First Nations children.[23] *The Behavioural Assessment Scale for Children, 3rd Edition (BASC-3)* is a 20-minute parent- and teacher-report of 2- to 21-year-old behavioural, emotional and social skills, as well as adaptive, attention and executive functioning in home, school and community settings.[13] Both parent- and teacher-report forms have excellent internal validity, very good test-retest reliability, and inter-rater reliability.[24]

Reference Tests were determined over a 6-month cross-site consultation process. Agreement on reliable and valid measures for 9 domains was achieved in six steps (see Table 2).

Table 2: Steps to establish cross-site agreement for reference tests

Step	Activity

For peer review only - http://bmjopen.bmj.com/site/about/guidelines.xhtml

1	An international survey of clinical practice broadly ascertained the standard assessment protocol for each domain at neurodevelopmental assessment clinics globally.
2	A systematic review outlined the standard assessment protocol for each domain when detecting impairment for an FASD diagnosis in internationally published research[25, 26]
3	Identification of tools from Step 1 and 2 with established Australian norms
4	A presentation to lead practitioners at each site introducing outcomes of Step 1, 2, and 3. Lead practitioners took information to their clinical team to compare with site specific assessment protocols for each domain
5	Monthly group meetings with lead practitioners across sites discussed pros and cons of preferred reference tests, until consensus was achieved.
6	When consensus could not be achieved on domain tests, the underlying construct of the domain was defined, and consensus was achieved on subtests measuring similar constructs. For example, for academic achievement, a valid and reliable reading subtest exists in both the Wide Range Achievement Test[27] and the Wechsler Individual Achievement Test.[28]

Table 3 outlines how the index tests correspond to reference tests selected for each neurodevelopmental domain used to diagnose FASD (excluding brain structure). All reference tests align with recommendations from the Australian Guide to the Diagnosis of FASD.[5]

Table 3: Index and reference tests used to assess neurodevelopmental domains

**Domain**	**Index Test**	**Reference Test**
Motor	RNDA: Gross motor and fine motor subscale	M-ABC 2 or BOT-2
Cognition	RNDA: Cognition subscale	WISC-V
Language	RNDA: Language subscale; BASC-3: Functional communication scale	CELF-5*
Academic Achievement	BASC-3: Learning problems scale	WIAT-III or WRAT-5 or WJ-IV
Memory	RNDA: Memory subscale	CMS: Stories and Dot Location

For peer review only - http://bmjopen.bmj.com/site/about/guidelines.xhtml

		subscales
Attention	RNDA: Behavior subscale (attention items); BASC-3: Attention Problems clinical scale	Connors-3 or Conners-4
Executive Function	BASC-3: Executive Functioning Index score	BRIEF-2
Affect Regulation	RNDA: Behavior subscale (affective items); BASC-3: Anxiety and Depression clinical scale	Previous diagnostic status (depression / anxiety)
Adaptive Behavior, Social Skills, Social Communication	RNDA: Self Care subscale; RNDA: Behavior subscale (peer play item); BASC-3: Adaptive Skills composite	ABAS-3 or Vineland-3

* equivalent language assessment validated for a First Nations population

Note:

- Rapid Neurodevelopmental Assessment: Australian Edition (RNDA)[13]
- Behaviour Assessment System for Children, 3rd Edition (BASC-3)[24]
- Movement Assessment Battery for Children: 2nd Edition (M-ABC 2)[29]
- Bruininks-Oseretsky Test of Motor Proficiency, 2nd Edition (BOT-2)[30]
- Wechsler Intelligence Scale for Children, Version 5 (WISC-V),[31] Australian and New Zealand Standardised Edition
- Clinical Evaluation of Language Fundamentals, Version 5 (CELF-5);[32]
- Wechsler Individual Achievement Test, 3rd Edition (WIAT-III)[28]
- Wide Range Achievement Test, 5th Edition (WRAT-5)[27]
- Woodcock-Johnson Achievement Test, 4th Edition, Australasian Adaptation (WJ-IV)[33]
- Children's Memory Scale (CMS)[34]
- Connors, 3rd Edition[35]
- Behaviour Rating Inventory of Executive Function, 2nd Edition (BRIEF-2)[36]
- Adaptive Behaviour Assessment System, 3rd Edition (ABAS-3)[37]
- Vineland Adaptive Behaviour Scales, 3rd Edition (Vineland-3)[38]

Procedure

Practitioners at each site will ask families with children who meet inclusion criteria if they consent to participate. Each site will administer index and reference tests as clinically appropriate. Index tests will be administered by trained practitioners including Aboriginal Health Workers, nurses, midwives, allied health, GPs and/or paediatricians (when available). Reference tests will be administered by allied health clinicians with relevant qualifications and credentialing for the tool (e.g., psychologists, occupational therapists, speech therapists), who have training and experience

For peer review only - http://bmjopen.bmj.com/site/about/guidelines.xhtml

For peer review only

diagnosing FASD, consistent with the Australian Guide to the Diagnosis of FASD.[5] Where possible, index tests and reference tests will be administered by different practitioners. Practitioners administering index tests will not have access to reference test results, and vice versa, until all tests are scored. When index or reference tests have been completed by third parties (e.g., the school), families will be asked for their consent to share data with participating study sites. A member of the research team will audit health records to extract test scores from completed assessments.

All practitioners and the research team will receive training in study protocols to ensure fidelity prior to administering tests and extracting data. Training will include: purpose of the project, rationale for test selection, and use of the RNDA. RNDA training includes studying standardised training videos developed by certified RNDA trainers, scoring training tapes, video recording assessments with a sample child, and a review of this video by a certified scorer (to ensure fidelity). If a site does not have practitioners available to complete a test, clinicians with relevant qualifications from the research team will administer the missing test. Check-in meetings with practitioners at each site will be held quarterly to ensure continued fidelity with study protocols. All index and reference tests aim to be completed within 9-months of the administration of the first test.

Phase II: Economic analysis

Study design

A cost-efficiency analysis will determine the cost and potential economic net benefits of: (a) implementing the rapid index tests compared to comprehensive reference tests, and (b) conducting assessments in primary healthcare compared to specialist services.

For peer review only - http://bmjopen.bmj.com/site/about/guidelines.xhtml

Primary and Secondary Outcomes

Economic analyses will be secondary outcomes, which include: the (1) direct healthcare costs, and (2) indirect costs of diagnosing FASD using index tests compared to reference tests; (3) the direct healthcare costs, and (4) indirect cost savings of delivering tests in primary healthcare compared to specialist clinics.

Participants

Two hundred families enrolled in Phase I will be asked about caregiver costs associated with index tests and reference tests. All sites will record health service costs associated with index tests and reference tests. Sites conducting comprehensive reference FASD assessments prior to the study will record health service costs of usual care for FASD assessments retrospectively.

Measures

(i) *Caregiver costs*

Direct and indirect assessment costs for each family including clinic fees, childcare, accommodation, and meals for assessment days will be surveyed from caregivers.

(ii) *Health service costs*

Prospective cost of delivering *index and reference tests* to participating children will be surveyed from practitioners and include: (1) discipline of the practitioner administering the test; (2) mode of delivery (e.g., clinic, home, school, or telehealth); (3) travel time and costs; (4) test administration time; and (5) indirect administration time for the assessment (e.g., scoring and interpretation).

For peer review only - http://bmjopen.bmj.com/site/about/guidelines.xhtml

Usual cost of *reference tests* will be collected from clinic managers in a structured interview, including time taken to administer and score each measure, travel time and other costs.

Procedure

Prior to participant recruitment, the cost of index and reference tests (usual care) for health services will be retrospectively collected in a structured phone/video interview from site managers. Site manager responses will be recorded, transcribed, and data will be extracted. Expected completion time for index and reference tests will also be extracted from test manuals. Families will be recruited and enrolled in the study as outlined in Phase I. Enrolled families and healthcare practitioners will complete surveys with one member from the research team that capture prospective cost information at the completion of testing. Therefore, the cost of index and reference tests will be collected retrospectively for tests administered as part of prior usual care from health service interviews, and costs will be collected prospectively for patients and health services recruited during the study period.

Patient and public involvement

The rapid assessment used in the current project forms part of a tiered approach to screening, diagnosing and supporting FASD in remote Australian communities that was co-designed with First Nations Elders. Co-design involved First Nations Elders, a community advisory group, First Nations remote health practitioners, clinical experts and researchers. The research questions in the current project originated from community advisory group because they asked, 'What is necessary and sufficient to screen for and diagnose FASD?' (ensuring judicious investment of resources), and

For peer review only - http://bmjopen.bmj.com/site/about/guidelines.xhtml

'Who in the health care system is best placed to diagnose FASD in a remote setting?' (reducing bottlenecks in specialist settings). Ongoing collaboration (focus groups, informal yarning, 3-monthly 1-week visits to community) between this group and the research team supported the development of the current protocol and this group has agreed to continue support for the duration of the project. Practitioners at new partner sites will be asked to identify a community representative interested in joining our advisory group. The advisory group will be informed of project progress and at the end of data collection, they will be involved in interpreting study results and will support dissemination of findings to local communities and the general public.

Data analysis

Phase I: Diagnostic accuracy

All data will be entered into a REDCap database hosted by Griffith University.[39] Data will be identifiable until all test results and survey data are matched to participants. Data will be de-identified for analysis. Pre-specified test positivity cutoffs reflecting clinical norms (e.g., T score>70) will be used, receiver operating curves will also be used to explore best possible cutoffs. Standard diagnostic statistics will be calculated, including: receiver operating curves, sensitivity, specificity, positive and negative predictive values, and likelihood ratios (using 95% confidence intervals).[40] Kappa statistics will be used to compare the reliability of the classification of children as normal/moderately impaired vs. severe on each measure of the neurodevelopmental domains.[41] Continuous scores are available for many of the assessments, which will be used to calculate correlations between matched scores from the index tests and reference tests. Bland-Altman plots will examine the agreement between matched index test and reference test scores, overall and by domain.[41] These plots will also help to

For peer review only - http://bmjopen.bmj.com/site/about/guidelines.xhtml

determine if any difference between the index and the reference is influenced by the actual scores on the tests (e.g., if the index-standard difference is larger when children have low or high scores). Diagnostic outcomes from the index tests will be compared to diagnostic outcomes from the reference tests to explore whether the index tests function as an effective screening tool for FASD.

Phase II: Economic analysis

A multivariable generalised linear model of the cost of diagnosis, with the predictive variable as the index test or reference test, will estimate the incremental cost between the two alternative diagnostic processes adjusting for differences in patient geographical location and other potential confounders. Sub-group cost analysis will explore heterogeneity of results between patients' subgroups by including an interaction term (e.g., location, diagnostic procedure) within the multivariable generalised linear model of cost. The expected value of implementation will be calculated as the net monetary benefit of the index tests (i.e., monetary benefits – costs) multiplied by the population of patients expected to benefit from the reference tests. Probabilistic and one way sensitivity analysis will be used to characterise the uncertainty in the economic results, highlighting the most important drivers for the value of implementing the index tests.

Summary

This research aims to establish the accuracy and cost-efficiency of rapid, culturally-responsive screening and assessment tools, which are part of a co-designed, tiered neurodevelopmental assessment process for diagnosing FASD in primary healthcare.[23, 42- 45] Diagnosing children with FASD in primary healthcare will lessen the burden on

For peer review only - http://bmjopen.bmj.com/site/about/guidelines.xhtml

specialist systems and help to place developmentally and socially/emotionally vulnerable children on support trajectories earlier. Our findings will help to demonstrate how primary healthcare, which is readily available to families regardless of their location, can support the assessment of FASD. Even partially adding information available to specialists can streamline subsequent interventions. Providing primary healthcare practitioners with the knowledge and tools to quickly identify children who are developmentally at-risk, to understand when to diagnose children, and when to seek specialist involvement, will reduce the demand on over-burdened specialist clinics. This will assist in providing timely access for assessment and treatment of children suspected of having FASD in Australia.

ETHICS AND DISSEMINATION

Ethics

The study has received ethical approval from the Griffith University Human Research Ethics Committee (2020/743) and Children's Health Queensland Hospital and Health Service Human Research Ethics Committee (HREC/20/QCHQ/63173). Consent to conduct research was obtained from the Kalkadoon Native Title Aboriginal Corporation RNTBC, representing the Traditional Owners of the lands from remote data collection sites.

Dissemination of findings

All study data will be archived on a secure server at Griffith University for at least five years after the last publication. Re-identifiable participant data will be collected on a secure web-based platform (REDCap) and exported to Griffith University servers for archiving. A summary report will be disseminated to participating site practitioners and

For peer review only - http://bmjopen.bmj.com/site/about/guidelines.xhtml

partner organisations. The community advisory group will support dissemination of outcomes within local communities. Outcomes of the project will be disseminated widely at national and international conferences, published in peer-reviewed journals and disseminated on social media.

Author contributions

DCS is the principal Chief Investigator, conceptualised the project, supervised the design and implementation of the research, and wrote the first draft of the protocol. EH conceptualised the project, planned the assessment protocols, data collection and analyses, and revised the protocol. MJZ-G and AJW participated in the project conceptualisation and design of the protocol, advise through membership on the steering committee, and contributed to preparation of the manuscript. JB and RSW advised on methodological and analytical aspects of the project, coordinate the health economics data collection and analyses, and critically revised the protocol. GS contributed to writing the first draft of the protocol and incorporating co-author revisions. WL contributed to project conceptualisation, manages project implementation, and revised the protocol. CW helped plan data collection, manages project data and storages procedures, and contributed to writing the protocol. SH, MP, DS, HT, IM, CH, KH, and AC are partner investigators on this project. They coordinate site project implementation, collect data, participate in monthly steering committee meetings and revised the manuscript. NR contributed to conceptualisation of this project, and revised the manuscript. KM, SR, KW and SR-T are chief investigators who participate in steering committee meetings and revised the manuscript.

Acknowledgements

For peer review only - http://bmjopen.bmj.com/site/about/guidelines.xhtml

We would like to acknowledge the many contributors to the DATAforFASD project. This includes the participating families, who graciously contribute extra time to complete lengthy assessments so that those in the future might get faster access to care. We thank the healthcare practitioners, who have added to their workload by incorporating this protocol into their existing clinical assessments. We thank the partner investigators, chief investigators, associate investigators and steering committee who have guided the conceptualisation and implementation of this project. We would like to thank our Community Advisory Group and remote health practitioners who started the journey to think differently about how to assess FASD so that more children could receive care close to home. We would like to thank Aunty Joan Marshall, Aunty Karen West, Theresa McDonald, Kara Rudken, Kerry Major, Michelle Parker-Tomlin, Keita Hada, Aunty Erica Buttigieg, Alexandrea Hofmann, Kerri O'Conner, Christopher-Lee Wesner, Viliame Sotutu. We are grateful for the support of policy influencers like Zena Martin and Daniel Williamson who have tirelessly advocated for our project. We are grateful for speech pathologists and community members who have helped co-design a language assessment that is best practice for remote First Nations communities. Specifically Jade Bethune, Claire Salter Parry, Bridgette Greathead, Grace Myatt, Bernadette Cantrall, Aunty Shirley Dawson, Taylor Wilson, Chris Doyle, and Benton Nemo. This project would not be possible without the support of our research team Heidi Webster, Jade Neuendorf, Amanda Amarrador, Caroline Gaderer, Tia Campbell, and Emma Harbeck.

Funding statement

This work was funded by an Australian National Health and Medical Research Council Partnership Grant GNT1170755.

For peer review only - http://bmjopen.bmj.com/site/about/guidelines.xhtml

Patient consent for publication

Not required.

Competing interests

The authors report that they have no competing interests, financial or otherwise.

Registration details for study

Trial Registration Number**:** ACTRN12622000498796

For peer review only

For peer review only - http://bmjopen.bmj.com/site/about/guidelines.xhtml

REFERENCES

1. Lange S, Probst C, Gmel G, *et al.* Global prevalence of fetal alcohol spectrum disorder among children and youth: a systematic review and meta-analysis. *JAMA Pediatr*. 2017;171:948-56.
2. May PA, Chambers CD, Kalberg WO, *et al.* Prevalence of fetal alcohol spectrum disorders in 4 US communities. *JAMA*. 2018;319:474-82.
3. Fitzpatrick JP, Latimer J, Carter M, *et al.* Prevalence of fetal alcohol syndrome in a population-based sample of children living in remote Australia: the Lililwan project. *J Paediatr Child Health*. 2015;51:450-7.
4. Popova S, Lange S, Probst C, *et al.* Estimation of national, regional, and global prevalence of alcohol use during pregnancy and fetal alcohol syndrome: a systematic review and meta-analysis. *The Lancet Global Health*. 2017;5:e290-e9.
5. Bower C, Elliott EJ, Zimmet M, *et al.* Australian guide to the diagnosis of foetal alcohol spectrum disorder: a summary. *J Paediatr Child Health*. 2017;53:1021.
6. McCleary K. Utilising a simulation-based teaching strategy to develop a new physiotherapy paediatric critical care service in a developing tertiary health service. *Aust Criti Care*. 2020;33:S17.
7. The Lowitja Institute. Close the gap: We nuture our culture for our future, and our culture nurtures us. In: Justice AaTSIS, editor. Australia: The Close the Gap Campaign Steering Committee; 2020.
8. Reid N, White C, Hawkins E, *et al.* Outcomes and needs of health and education professionals following fetal alcohol spectrum disorder-specific training. *J Paediatr Child Health*. 2020;56:317-23.
9. Bossuyt PM, Reitsma JB, Bruns DE, *et al.* STARD 2015: an updated list of essential items for reporting diagnostic accuracy studies. *Clinical Chem*. 2015;61:1446-52.
10. Bush K KD, McDonell MB, *et al.* The audit alcohol consumption questions (AUDIT-C): an effective brief screening test for problem drinking. *Arch Intern Med*. 1988;158:1789-95.
11. Bradley KA, Bush KR, Epler AJ, *et al.* Two brief alcohol-screening tests from the alcohol use disorders identification test (AUDIT-C): validation in a female veterans affairs patient population. *Arch Intern Med*. 2003;163:821-9.
12. Hemingway SJA, Bledsoe JM, Brooks A, *et al.* Comparison of the 4-digit code, Canadian 2015, Australian 2016 and Hoyme 2016 fetal alcohol spectrum disorder diagnostic guidelines. *Adv in Pediatric Resear*. 2019;6.
13. Khan N, Muslima H. Rapid neurodevelopmental assessment of children: a practical guide for multidisciplinary professionals. Dhaka: Bangladesh Protibondhi Foundation; 2017.
14. Reynolds C, Kamphaus R. Behaviour assessment system for children – third edition manual: Circle Pines, MN: American Guidance Service; 2015.
15. Anggun T. S. T., Kraska J, Bell K., *et al.* Confirmatory factor analyses of the behavior assessment system for children – third edition among an australian sample. *Edu & Dev Psych*. 2021;38:131-42.
16. Muslima H, Khan NZ, Shilpi AB, *et al.* Validation of a rapid neurodevelopmental assessment tool for 10- to 16-year-old young adolescents in Bangladesh. *Child: Care, Health and Dev*. 2016;42:658-65.
17. Thompson L, Peñaloza RA, Stormfields K, *et al.* Validation and adaptation of rapid neurodevelopmental assessment instrument for infants in Guatemala. *Child: Care, Health and Dev*. 2015;41:1131-9.

For peer review only

18. National Rural Health Alliance I. Mental health in rural and remote Australia: Fact sheet. 2017.
19. Flannigan KR, Coons-Harding KD, Turner O, *et al.* A survey of measures used to assess brain function at fasd clinics in Canada. *Canadian Psych*. 2022;63:106-19.
20. Khan NZ, Muslima H, Begum D, *et al.* Validation of rapid neurodevelopmental assessment instrument for under-two-year-old children in Bangladesh. *Pediatr*. 2010;125:e755-e62.
21. Khan NZ, Muslima H, Shilpi AB, *et al.* Validation of rapid neurodevelopmental assessment for 2-to 5-year-old children in Bangladesh. *Pediatr*. 2013;131:e486-e94.
22. Khan NZ, Muslima H, El Arifeen S, *et al.* Validation of a rapid neurodevelopmental assessment tool for 5 to 9 year-old children in Bangladesh. *Pediatr*. 2014;164:1165-70. e6.
23. Reid N, Page M, McDonald T, *et al*. Integrating cultural considerations and developmental screening into an Australian first nations child health check. *Australian J of Primary Health*. 2022.
24. Kamphaus RW, Reynolds CR. Behavioral and emotional screening system (BASC): Manual. Pearson Inc.; 2007.
25. Reid N, Shanley DC, Logan J, *et al.* International survey of specialist fetal alcohol spectrum disorder diagnostic clinics: comparison of diagnostic approach and considerations regarding the potential for unification. *Inter J Environ Resear and Public Health*. 2022;19:15663.
26. Reid N, Hawkins E, White C, *et al.* Is a unified approach to fasd diagnosis possible? Results of an international surveyof diagnostic clinics. In: Alcoholism-clinical experimental research. NJ, USA: Willey; 2021.
27. Wilkinson GS, J. RG. Wide range achievement test, fifth edition (WRAT-5). 2017.
28. Wechsler D. Wechsler individual achievement test (3rd ed.). San Antonio, TX: Pearson; 2009.
29. Henderson SE, Sugden D, Barnett AL. Movement assessment battery for children-2. *Resear in Dev Disabilities*. 2007.
30. Bruininks RH. Bruininks-oseretsky test of motor proficiency: BOT-2: NCS Pearson/AGS Minneapolis, MN; 2005.
31. Terriot K, Ozenne R. Wechsler intelligence scale for children, v version (WISC-v). ANAE - Approche Neuropsychologique des Apprentissages chez l'Enfant. 2015;27:95-102.
32. Wiig EH, Semel E, Secord WA. Clinical evaluation of language fundamentals-fifth edition (CELF-5). Bloomington, MN: NCS Pearson; 2013.
33. Schrank FA, McGrew KS, Mather N. Woodcock johnson fourth edition - australasian adaptation Rolling Meadows, IL: Riverside; 2014.
34. Cohen MJ. Children’s memory scale. In: Kreutzer JS, DeLuca J, Caplan B, editors. Encyclopedia of clinical neuropsychology. New York, NY: Springer New York; 2011. p. 556-9.
35. Conners CK, Pitkanen J, Rzepa SR. Conners 3rd edition. In: Kreutzer JS, DeLuca J, Caplan B, editors. Encyclopedia of clinical neuropsychology. New York, NY: Springer New York; 2011. p. 675-8.
36. Gioia GA, Isquith PK, Guy SC, *et al.* Test review behavior rating inventory of executive function. *Child Neuropsy*. 2000;6:235-8.
37. Harrison PL, Oakland T. Adaptive behavior assessment system: Psychological Corporation San Antonio, TX; 2000.

For peer review only

38. Sparrow SS, Cicchetti DV, Saulnier CA. Vineland adaptive behavior scales, third edition. Bloomington, MN: NCS Pearson, Inc.; 2016.
39. Patridge EF, Bardyn TP. Research electronic data capture (REDCap). *J Med Library Asso: JMLA*. 2018;106:142.
40. Chrobak K. Studying a study & testing a test: Reading evidence-based health research 6th ed. *J Canadian Chiro Asso*. 2013;57:96-.
41. Morgan CJ, Aban I. Methods for evaluating the agreement between diagnostic tests. Springer; 2016.
42. Shanley DC, Hawkins E, Page M, *et al.* Protocol for the yapatjarrathati project: a mixed-method implementation trial of a tiered assessment process for identifying fetal alcohol spectrum disorders in a remote australian community. *BMC Health Services Research*. 2019;19:1-11.
43. Reid N, Page M, McDonald T, *et al.* Integrating cultural considerations and developmental screening into an australian first nations child health check. *Aust J Primary Health*. 2022.
44. Reid N, Hawkins E, Liu W, *et al.* Yarning about fetal alcohol spectrum disorder: outcomes of a community-based workshop. *Resear in Dev Disabilities*. 2021;108:103810.
45. Miller L, Shanley DC, Page M, *et al.* Preventing drift through continual co-design: refining the prototype of a tiered fasd assessment. *Inter J Environ Resear and Public Health*. 2022;18.

For peer review only

Section & Topic	No	Item	Reported on page #
**TITLE OR ABSTRACT**			
	**1**	Identification as a study of diagnostic accuracy using at least one measure of accuracy (such as sensitivity, specificity, predictive values, or AUC)	1
**ABSTRACT**			
	**2**	Structured summary of study design, methods, results, and conclusions (for specific guidance, see STARD for Abstracts)	2-3
**INTRODUCTION**			
	**3**	Scientific and clinical background, including the intended use and clinical role of the index test	6
	**4**	Study objectives and hypotheses	6
**METHODS**			
Study design	**5**	Whether data collection was planned before the index test and reference standard were performed (prospective study) or after (retrospective study)	7
Participants	**6**	Eligibility criteria	8
	**7**	On what basis potentially eligible participants were identified (such as symptoms, results from previous tests, inclusion in registry)	8
	**8**	Where and when potentially eligible participants were identified (setting, location and dates)	8
	**9**	Whether participants formed a consecutive, random or convenience series	8
Test methods	**10a**	Index test, in sufficient detail to allow replication	10-11
	**10b**	Reference standard, in sufficient detail to allow replication	11-12
	**11**	Rationale for choosing the reference standard (if alternatives exist)	12-13
	**12a**	Definition of and rationale for test positivity cut-offs or result categories of the index test, distinguishing pre-specified from exploratory	17
	**12b**	Definition of and rationale for test positivity cut-offs or result categories of the reference standard, distinguishing pre-specified from exploratory	17
	**13a**	Whether clinical information and reference standard results were available to the performers/readers of the index test	13
	**13b**	Whether clinical information and index test results were available to the assessors of the reference standard	13
Analysis	**14**	Methods for estimating or comparing measures of diagnostic accuracy	17
	**15**	How indeterminate index test or reference standard results were handled	Protocol (no data)
	**16**	How missing data on the index test and reference standard were handled	Protocol (no data)
	**17**	Any analyses of variability in diagnostic accuracy, distinguishing pre-specified from exploratory	Protocol (no data)
	**18**	Intended sample size and how it was determined	8-9
**RESULTS**			
Participants	**19**	Flow of participants, using a diagram	Protocol (no data)
	**20**	Baseline demographic and clinical characteristics of participants	9 (planned)
	**21a**	Distribution of severity of disease in those with the target condition	Protocol (no data)
	**21b**	Distribution of alternative diagnoses in those without the target condition	Protocol (no data)
	**22**	Time interval and any clinical interventions between index test and reference standard	14
Test results	**23**	Cross tabulation of the index test results (or their distribution) by the results of the reference standard	17
	**24**	Estimates of diagnostic accuracy and their precision (such as 95% confidence intervals)	17
	**25**	Any adverse events from performing the index test or the reference standard	19
**DISCUSSION**			
	**26**	Study limitations, including sources of potential bias, statistical uncertainty, and generalisability	18
	**27**	Implications for practice, including the intended use and clinical role of the index test	18
**OTHER INFORMATION**			
	**28**	Registration number and name of registry	21

For peer review only

	**29**	Where the full study protocol can be accessed	This is the full study protocol
	**30**	Sources of funding and other support; role of funders	20

For peer review only

STARD 2015

AIM

STARD stands for “Standards for Reporting Diagnostic accuracy studies”. This list of items was developed to contribute to the completeness and transparency of reporting of diagnostic accuracy studies. Authors can use the list to write informative study reports. Editors and peer-reviewers can use it to evaluate whether the information has been included in manuscripts submitted for publication.

EXPLANATION

A **diagnostic accuracy study** evaluates the ability of one or more medical tests to correctly classify study participants as having a **target condition.** This can be a disease, a disease stage, response or benefit from therapy, or an event or condition in the future. A medical test can be an imaging procedure, a laboratory test, elements from history and physical examination, a combination of these, or any other method for collecting information about the current health status of a patient.

The test whose accuracy is evaluated is called **index test.** A study can evaluate the accuracy of one or more index tests. Evaluating the ability of a medical test to correctly classify patients is typically done by comparing the distribution of the index test results with those of the **reference standard**. The reference standard is the best available method for establishing the presence or absence of the target condition. An accuracy study can rely on one or more reference standards.

If test results are categorized as either positive or negative, the cross tabulation of the index test results against those of the reference standard can be used to estimate the **sensitivity** of the index test (the proportion of participants *with* the target condition who have a positive index test), and its **specificity** (the proportion *without* the target condition who have a negative index test). From this cross tabulation (sometimes referred to as the contingency or “2x2” table), several other accuracy statistics can be estimated, such as the positive and negative **predictive values** of the test. Confidence intervals around estimates of accuracy can then be calculated to quantify the statistical **precision** of the measurements.

If the index test results can take more than two values, categorization of test results as positive or negative requires a **test positivity cut-off**. When multiple such cut-offs can be defined, authors can report a receiver operating characteristic (ROC) curve which graphically represents the combination of sensitivity and specificity for each possible test positivity cut-off. The **area under the ROC curve** informs in a single numerical value about the overall diagnostic accuracy of the index test.

The **intended use** of a medical test can be diagnosis, screening, staging, monitoring, surveillance, prediction or prognosis. The **clinical role** of a test explains its position relative to existing tests in the clinical pathway. A replacement test, for example, replaces an existing test. A triage test is used before an existing test; an add-on test is used after an existing test.

Besides diagnostic accuracy, several other outcomes and statistics may be relevant in the evaluation of medical tests. Medical tests can also be used to classify patients for purposes other than diagnosis, such as staging or prognosis. The STARD list was not explicitly developed for these other outcomes, statistics, and study types, although most STARD items would still apply.

DEVELOPMENT

This STARD list was released in 2015. The 30 items were identified by an international expert group of methodologists, researchers, and editors. The guiding principle in the development of STARD was to select items that, when reported, would help readers to judge the potential for bias in the study, to appraise the applicability of the study findings and the validity of conclusions and recommendations. The list represents an update of the first version, which was published in 2003.

More information can be found on http://www.equator-network.org/reporting-guidelines/stard.

For peer review only
